# Microdroplet Synthesis of Silver Nanoparticles with Controlled Sizes

**DOI:** 10.3390/mi10040274

**Published:** 2019-04-24

**Authors:** Tingting Hong, Aijuan Lu, Wenfang Liu, Chuanpin Chen

**Affiliations:** 1 School of Pharmaceutical Sciences, Central South University, Changsha 410013, China; hongtingting0203@163.com (T.H.); liuwenfang@csu.edu.cn (W.L.); 2 Hunan Key Laboratory Cultivation Base of the Research and Development of Novel Pharmaceutical Preparations, Changsha 410219, China; 147211005@csu.edu.cn

**Keywords:** silver nanoparticle, microchip, microdroplet

## Abstract

A method was developed to synthesize silver nanoparticles with controlled size and Localized Surface Plasmon Resonance (LSPR) wavelength. In a microchip, droplets with high monodispersity and stability were produced. Using droplets as microreactors, seed-mediated growth approach was successfully applied for silver nanoparticles preparation. It was observed that nanoparticles size and LSPR wavelength could be optimized via adjusting synthesis conditions, such as droplets heating temperature, reaction time, and concentration of silver seeds and silver nitrate in aqueous phase. These results indicated that the proposed microdevices could provide a convenient and inexpensive approach for preparing nanoparticles with optimum properties.

## 1. Introduction

Since at least one dimension of silver nanocrystals decreases to the range of 1–100 nm, the optical, electronic, catalytic and chemical properties of silver nanoparticles are quite different from bulk metal [1]. Silver nanoparticles have received considerable attention during the past decades due to their unique properties. For instance, with optical properties of localized surface plasmon resonance (LSPR), silver nanostructures were used as substrates for surface-enhanced Raman scattering (SERS), contrast agents for bioimaging, and biosensors [2]. The intrinsic properties of a silver nanoparticle are mainly depended on particles size, shape, composition, crystallinity, and structure, among which size and shape are more significant [3]. Theoretically, the properties of silver nanoparticle can be regulated by controlling one of these parameters [4]. For example, the high enhancement effect is achieved with hundreds of nanometer-sized particles in SERS [5]; while, the optimal size of silver nanoparticles is about 50 nm for nonresonant SERS [6]. Therefore, achieving well-controlled size and shape is a fundamental requirement for the synthesis of silver nanoparticles with desirable properties.

In spite of the importance of controlled synthesis, the synthesis of silver nanoparticles with controllable size is a challenge. Varieties of methods have been reported to prepare size-controlled silver nanostructures. The classical methods can be classified into two categories: seed-mediated synthesis and photochemical synthesis. Wan et al. synthesized quasi-spherical silver nanoparticles with tunable size by following a controlled seed-mediated growth approach via thermal reduction of silver nitrate with citrate [7]. Moreover, Bastús et al. synthesized silver nanoparticles by using a combination of two chemical reducing agents, sodium citrate and tannic acid [8]. The size of silver nanoparticles was controlled via adjusting reaction temperature, pH, and the concentration of reducing agents and seed. As a result, citrate-coated spherical silver nanoparticles with controllable size from 10 to 200 nm were obtained. However, it is complicated to achieve large-sized silver nanoparticles in most similar methods, because stepwise seeding growth is needed. The size of silver nanocrystals prepared via photochemical pathway was mainly affected by the irradiating time, intensity and wavelength [9,10]. However, the wavelength of light source available in the laboratory is limited, limiting the synthesis of silver nanocrystals with controlled size in a wide range, and silver nanostructure shape can change during the irradiation process [11,12,13]. In addition, template-based method is also commonly used to synthesize size-controlled silver nanowires or nanorods by the steric and structure-directing effect. The templates are mesoporous materials, carbon nanotubes, polymer materials, and micelles [14]. However, the structures of templates, especially for polymer and biological templates, are highly sensitive to the surroundings, and impurities may be introduced during the process of removing templates.

Another rapidly developing research filed in recent years is droplet microreactors based on microfluidic technology. Droplets can precisely control entire nanoparticles preparation processes. Moreover, the micro-scale size of droplets facilitates the achievement of rapid heat and mass-transfer rates. Droplet microreactors have been proved a powerful tool for biological and chemical reactions [15,16]. For example, colloidal nanoparticles were synthesized via the droplet microreactors [17]. Compared with the conventional synthesis in large reaction vessel, droplet microreactors possess the following advantages [18,19]. First, the consumption of reagents is small during the process of optimizing reduction condition, especially, for expensive or toxic reagents. Second, the enhanced mass and heat transfers due to the large specific surface area, contribute a better control of reaction concentration and temperature, which govern particle size and size distribution. Third, scaling up the production of nanoparticles can be achieved just by extending the duration of the synthesis, without adjusting other experimental parameters, which not scale linearly with the volume of the reaction solution [20]. Moreover, droplet microreactors can eliminate concentration dispersion in single phase microreactors and avoid the fouling by reagents or nanocrystal contacting with the inner of channel. These features make droplet microreactors uniquely suitable for synthesizing size-controlled nanomaterials.

There have been many reports about the synthesis of metal nanoparticles in droplet-based microreactors. Additionally, Pa, Pd, Au, and Ag nanocrystals with controlled sizes and shapes were synthesized in droplet-based microreactor by the group of Xia [21,22,23]. Some researchers investigated the controlled synthesis of silver nanoparticles in microdroplet reactors. Xia et al. synthesized silver nanocubes with controlled sizes in droplet microreactors for the first time [24,25,26]. The edge lengths of silver nanocubes can be tuned in the range of 30 to 100 nm by changing the reaction time, the amount of silver nitrate, and the amount of silver seeds in the droplets. To prepare silver nanoparticles with controlled size, we constructed microdroplet reactors. Herein, a method of seed-mediated growth was developed, and silver nitrate was reduced by sodium citrate on the surface of silver seeds. The size of obtained silver nanoparticles and the color of the sol could be easily changed by varying the reaction time, heating temperature, and concentration of silver seeds and silver precursors. For instance, nanoparticles size increased and LSPR wavelength bathochromic shifted when the temperature increased (40–80 °C). The LSPR wavelength bathochromic shifted with the increase of droplets heating time or concentration of silver nitrate. This work provided a novel way for synthesizing nanomaterials with high quality.

## 2. Experimental Section

### 2.1. Materials

All the used chemicals were analytical grade and used as received from their corresponding suppliers, unless otherwise noted. Silver nitrate (AgNO_3_, ≥99.8%) and sodium borohydride (NaBH_4_, 96.0%) were purchased from Sinopharm Chemical Reagent Co., Ltd. (Shanghai, China). Trisodium citrate dihydrate (Na_3_C_6_H_5_O_7_·2H_2_O, 99.0%) and liquid paraffin were obtained from Tianjin Fengchuan Chemical Reagent Co., Ltd. (Tianjin, China). Sorbitanmonooleate (Span 80) was provided by Tianjin Kemiou Chemical Reagent Co., Ltd. (Tianjin, China). Sylgard 184 silicone elastomer kits including polydimethylsiloxane (PDMS) and a crosslinking agent were purchased from Dow Corning Co. (San Diego, CA, USA). Ultrapure deionized water with resistance of 18 MΩ·cm (filtration system: KL-MINI4-T, Kertone Water Treatment Co., Ltd., Kertone, UK) was used for all experiments.

### 2.2. Silver Seed Synthesis

The silver seed were prepared by using the method reported by Jia et al. In brief, nearly spherical silver nanoparticle seeds (~4 nm on average) were synthesized by drop-wise adding 0.5 mL freshly prepared sodium borohydride solution (50 mM) to a mixture of aqueous silver nitrate (50 mL of 0.1 mM) and TSC (0.5 mL of 30 mM) solution under vigorous stirring.

### 2.3. Microfluidic Device Fabrication

Photolithographic technique was used to fabricate a SU-8 photoresist mold on a silicon wafer (Delta 80, Suss MicroTec, Germany). A mixture of PDMS oligomer and crosslinking agent (10:1, w/w) was cast onto the mold to form a replication, then it was degassed in a vacuum chamber for 30 min and heated in an oven for 2 h at 60 °C for the polymer to cure. After curing, a PDMS replication was removed from the mold and was drilled at the channel inlets. The patterned surface of PDMS replication was treated with corona discharge for 1 min, and then directly bonded to a 2-mm-thick blank PDMS plate. The bonded device was then baked at 60 °C for at least 2 h in an oven.

### 2.4. Seed-Mediated Growth of AgNPs in Microdroplet Reactors

**Figure 1:**
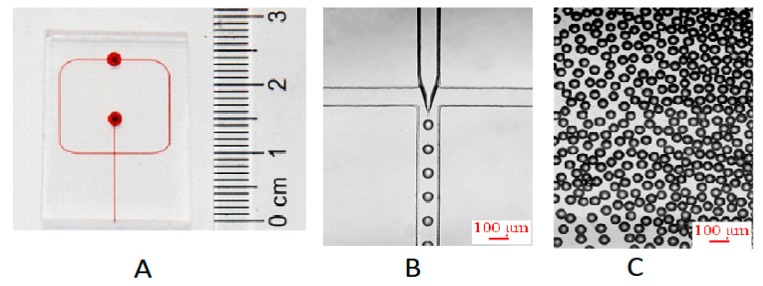
(**A**) Photograph of the polydimethylsiloxane (PDMS) microchip. Channel and reservoirs were full of red ink for visualization. (**B**) Microphoto of the process of microdroplets preparation at cross-junction channel. The velocity of aqueous phase and oil phase was 14 and 80 μL/min, respectively. (**C**) Microphoto of microdroplets off chip.

A flow focusing microchip (Figure 1A) was used to produce uniform microdroplets containing silver seeds and growth solution at first. The width and height of the microchannel were 100 μm and 100 μm, respectively. Oil phase (continuous phase) and aqueous phase (dispersed phase) were delivered by two syringe pumps (Harvard, PHD 2000) into the microchip to produce microdroplets. In a standard synthesis, a mixture solution of AgNO_3_ (5.0 mM, 3.6 mL), TSC (30.0 mM, 6.0 mL), silver seeds (1.0 mL) and pure water (7.4 mL) serving as aqueous phase was introduced into *I*1 at a flow velocity of 14 μL/min, while liquid paraffin with 1.5% (w/w) Span 80 used as oil phase was introduced into *I*2 at a flow velocity of 80 μL/min. Polytetrafluoroethylene (PTFE) tubes with an inner diameter of 0.5 mm were used for connecting the syringes to the microchip inlets, and for transferring the product microdroplets from the microchip outlet to a 50 mL glass vial to collect microdroplets.

The collected microdroplets containing reactants were then heated at 60 °C for a period of time to ensure silver seed grow up within microdroplets. During the heating process, aliquots of microdroplets were taken out from glass vial and were centrifuged at 8000 r/min for 10 min to separate aqueous phase from microdroplets, and then at 15,000 r/min for 10 min to precipitate Ag particles from aqueous phase. The as-obtained Ag particles were washed with ethanol once and twice with deionized water before dispersed in deionized water by ultrasonication.

### 2.5. Characterizations

The optical properties of the obtained silver colloidal solutions were characterized by using UV-Vis spectrometer (UV-2450, SHIMADZU) with a resolution of 0.5 nm.

## 3. Results and Discussion

### 3.1. Formation of Microdroplets

**Figure 2:**
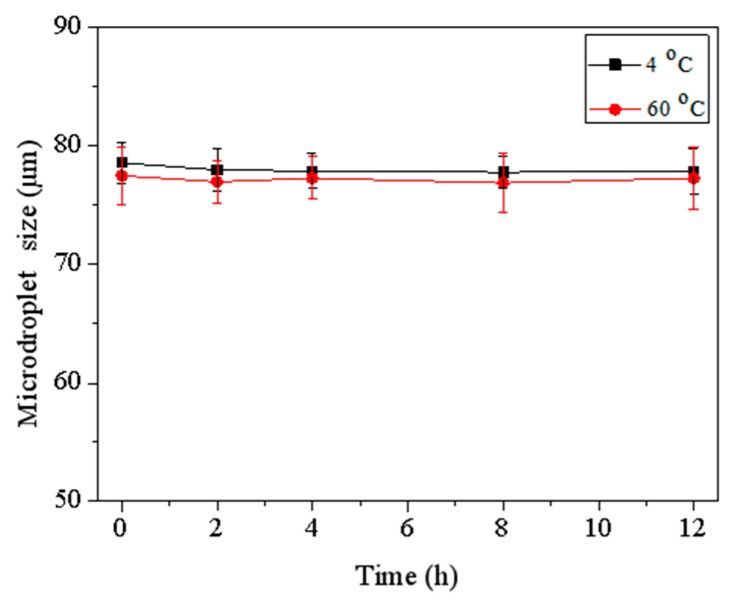
Relationship between size of microdroplets and time at 4 °C and 60 °C.

In this microfluidic method to synthesize silver nanoparticles, we first produced microdroplets of the initial reaction mixture. Figure 1B shows the process of microdroplets formation in the cross-junction channel of microchip. When aqueous phase encountered water-immiscible oil phase at the cross-junction of channel, the aqueous stream instantly broke up into microdroplets under the combined effect of shear forces and surface tension between continue phase and dispersed phase, surrounded and separated by the oil phase. Under stable fluidic conditions, uniform microdroplets with equal sizes were formed. The microdroplets could keep stable and did not coalesce in the channel of microchip and off microchip (see Figure 1C) because the concentration of Span 80 was 1.5 wt %, higher than its critical micelle concentration (CMC) (1.13 × 10^−2^ wt %). To obtain Ag particles with a narrow distribution, the microdroplets need to be uniform and independent of each other, do not coalesce during the long time for silver seed to grow. Before microdroplets were broken by centrifugation to separate Ag particles, microdroplets were collected and stored at 4 °C and heated at 60 °C. Figure 2 shows that the size of microdroplets changed a little during those processes for a long time. Therefore, the microdroplets provided a stable environment for the synthesis of silver nanoparticles. In addition, the volume of microdroplets can be controlled by changing flow velocity of aqueous phase and oil phase or by changing the geometric construction of channel. Additionally, scaling up the production can be easily realized by extending microdroplets collection time. Compared with segmented flow reactors, the proposed microdroplets reactors could wrap all the reagents and reaction products without the possibility of contamination.

### 3.2. Formation of Ag Colloidal in Microdroplets

**Figure 3:**
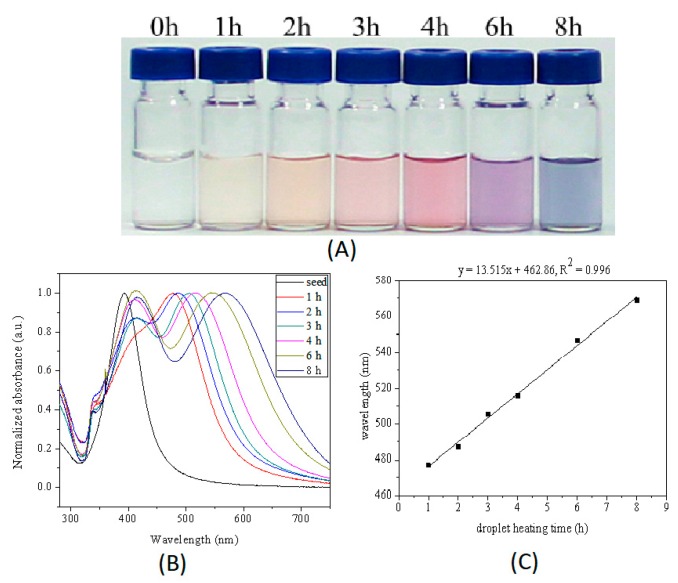
(**A**) Photograph and (**B**) Normalized UV-Vis extinction spectra of series of as-prepared samples obtained being heated at 0, 1, 2, 3, 4, 6, and 8 h. (**C**) Relationship between the in-plane dipole plasmon resonance peak of nanoparticles and the heating time. The initial reactants concentration in microdroplets was 0.5 mM for silver nitrate, 5.0 mM for sodium citrate, and 66 μL/mL for seeds. The size of microdroplets was ca. 70 μm. The reaction temperature was 60 °C.

Silver seeds could grow up in the microdroplets by reduction absorbed silver ions at the surface of seed with sodium citrate at 60 °C. Sodium citrate is a mild reducing agent commonly used to synthesize silver or gold nanoparticles at elevated temperature, for example, 100 °C was used in Lee-Meisel method to synthesize silver nanoparticles. In this work, the silver nanoparticles were synthesized by the method of seed-based thermal reduction in microdroplets at 60 °C, a relatively mild condition. As shown in Figure 3A, color of aqueous phase turned from nearly colorless to purple after microdroplets containing a mixture solution of silver seeds, silver nitrate and sodium citrate were heated at 60 °C for 6 h, indicating the large silver nanoparticles were obtained.

### 3.3. Size Tunability of Silver Nanoparticles

The color of the silver sol could be tuned simply by changing the microdroplets heating time. With the increase of microdroplets heating time, the color of silver sol changed from colorless to yellow and purple (see Figure 3A). The changes of color reflect changes in the LSPR bands. The seed-mediated growth process of silver nanoparticles was characterized by ultraviolet-to-visible (UV-Vis) spectra of silver nanoparticles synthesized at different microdroplets heating time. The microdroplets heating time is the reaction time for seeds to grow up. Normalized UV-Vis extinction spectrum of Ag nanoparticles synthesized with different heating time are shown in Figure 3B. UV-Vis spectrum of most of those products presented three peaks located at approximately 330 nm (weak shoulder peak), 410 nm and 470 nm. The three peaks could be attributed to the out-of-plane quadrupole, the out-of-plane dipole, and the in-plane dipole Surface Plasmon Resonance (SPR) modes of a triangular nanoplate, respectively. The in-plane dipole SPR wavelength position of silver nanoparticles bathochromic shifted from 395 to 553 nm with the increase of microdroplets heating time. The in-plane dipole plasmon resonance wavelength was very sensitive to the nanoparticle size and shape, and many literatures reported that the major LSPR peak bathochromic shifted with the increase in the nanosphere or nanoplate size. We could assume that the silver nanoparticles grew with the increase of microdroplets heating time. Compared with the UV-Vis spectrum of seeds, the UV-Vis spectra of products obtained at 1 h and 2 h had one broad absorption peak but bathochromic shifted from the wavelength position of silver seed, indicating the shape of products synthesized with 1 h and 2 h might have little difference with seeds except the size of products was bigger than that of seeds. The absorbance at ~410 nm of products synthesized under 60 °C for 3 h, 4 h, and 6 h were larger than absorbance at wavelength of in-plane dipole plasmon resonance of corresponding products. These results could be partly attributed to the presence of small spherical silver nanoparticles.

**Figure 4:**
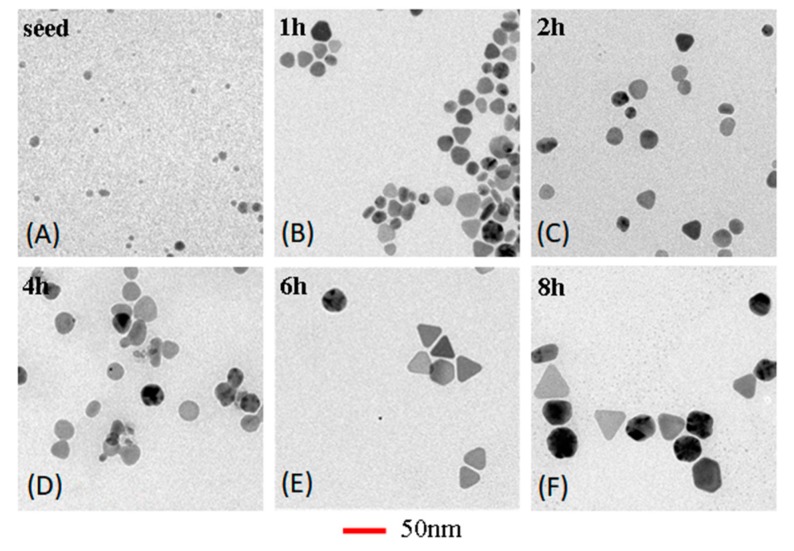
TEM image of AgNPs synthesized with different reaction time: (**A**) 0 h, (**B**) 1 h, (**C**) 2 h, (**D**) 4 h, (**E**) 6 h, and (**F**) 8 h. The experiment conditions were the same as Figure 3.

The obtained Ag nanoparticles synthesized at different heating time were further characterized by TEM. Figure 4 shows TEM images of the silver nanoparticles with sizes increasing from 6.2 to 16.4, 17.4, 23.1, 27.2 and 34.2 nm that were obtained by heating microdroplets at 60 °C for 0, 1, 2, 4, 6, and 8 h. Therefore, the size of silver nanoparticles increased with increasing the microdroplets heating time. The results suggested that microdroplets heating time had a prominent effect on silver nanoparticles size due to the Ostwald ripening. In addition, size distribution also enlarged with increasing time. Terminating the synthesis at different microdroplets heating time could provide a convenient and precise method to control the size of silver nanoparticles over a broad range. The analysis result of UV-Vis spectra was consistent with TEM result. Moreover, the in-plane dipole surface plasmon band has been proved to be a good indicator of general silver nanoparticles. Therefore, we could assess the effect of reaction parameters on the morphology of nanoparticles by analyzing the UV-Vis spectra of obtained silver particles.

**Figure 5:**
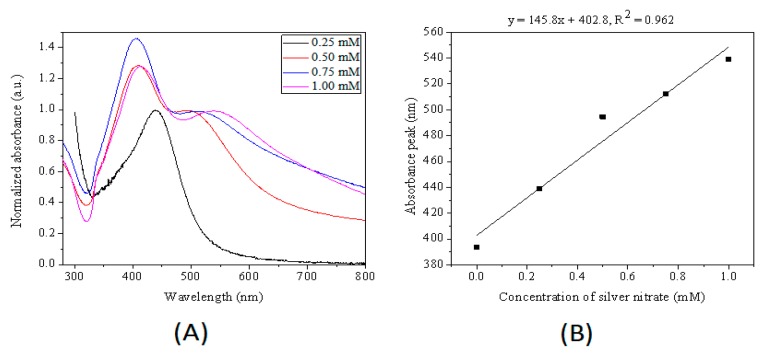
(**A**) Normalized UV−Vis extinction spectra of samples obtained by varying initial concentration of silver nitrate in the droplets. (**B**) Relationship between the in-plane dipole plasmon resonance peak of product nanoparticles and the silver citrate concentration. The initial concentration of silver nitrate was 0.25, 0.50, 0.75, 1.0 mM. The amount of seeds for four aqueous phases were 66 μL/mL. The size of microdroplets was ca. 70 μm. The microdroplets were heated at 60 °C for 4 h.

Concentration of silver ions also play an important role in controlling the size of silver nanoparticles. Figure 5 shows that one SPR peak located at 439 nm for silver nanoparticles synthesized with 0.25 mM silver nitrate and two LSPR bands for silver nanoparticles synthesized with 0.5, 0.75 and 1 mM silver nitrate. The in-plane dipole resonance bands of silver nanoparticles bathochromic shifting from 497 to 513, and 540 nm were synthesized with 0.5, 0.75 and 1 mM silver nitrate, respectively. These results indicated that an increase of silver cations concentration caused an increase in silver nanoparticles size. The reaction rate was fast for high concentration of silver nitrate since silver cations were essential reactant for the synthesis of silver nanoparticles, leading to larger size of silver nanoparticles within the same reaction time.

**Figure 6:**
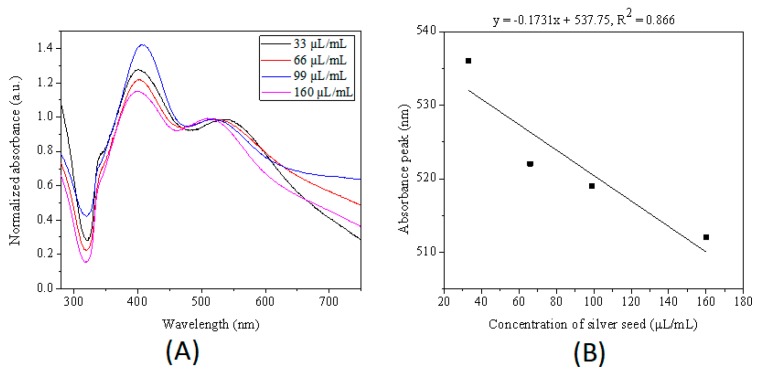
(**A**) Normalized UV−Vis extinction spectra of products obtained by adding different amount of seed in the droplets. (**B**) Relationship between the in-plane dipole plasmon resonance peak of product nanoparticles and the concentration of seed. The initial reactants concentration in microdroplets was 1 mM for silver nitrate, 10 mM for sodium citrate, and different amount of seeds. The size of microdroplets was ca. 70 μm. The microdroplets were heated at 60 °C for 6 h.

The amount of added seed can alter the size of final products as well. To investigate the influence of the concentration of seed, the silver nanoparticles were synthesized in microdroplets by reducing silver nitrate with sodium citrate at the surface of added silver seeds with various concentrations. As shown in Figure 6, the major plasmon band of Ag nanoparticles gradually blue shifted from 538 to 521, 519 and 512 nm as the seeds concentration increased from 33 to 66, 99, and 160 uL/mL, suggesting that the average size of silver nanoparticles decreased with the increase of silver seeds number in the initial reaction solution. A similar result was reported by Jana et al. and Xia et al.

**Figure 7:**
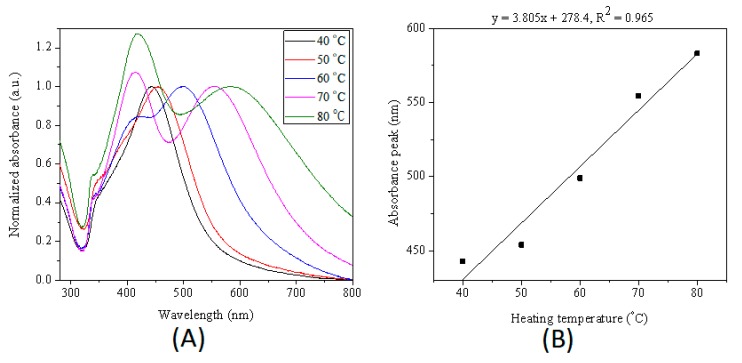
(**A**) Normalized UV−Vis extinction spectra of products extracted from droplets heated at 40, 50, 60, 70 and 80 °C for 4 h. (**B**) Relationship between the in-plane dipole plasmon resonance peak of product nanoparticles and heating temperature. The initial reactants concentration in microdroplets was 0.5 mM for silver nitrate, 5 mM for sodium citrate, and 66 μL/mL for seeds. The size of microdroplets was ca. 70 μm.

Since sodium citrate can reduce silver nitrate at elevated temperature, the reaction temperature plays a very important role in controlling size of silver nanoparticles. Figure 7 shows that just one SPR peak for silver nanoparticles synthesized at 40 and 50 °C, and two LSPR peaks for silver nanoparticles synthesized at 60, 70 and 80 °C. The in-plane dipole resonance bands of silver nanoparticles synthesized at different reaction temperatures bathochromic shifted from 498.5 nm (60 °C) to 553.5 nm (70 °C) and 584 nm (80 °C). Additionally, the peaks located at ~410 nm became intense with the increase of reaction temperature in the range of 60 to 80 °C. When the reaction temperature was not high enough to generate silver seed within 4 h, only growth of initial seed was happened. When the temperature was lower than nucleation temperature, the higher the temperature, the faster the reduction rate, leading to large size silver nanoparticles.

## 4. Conclusions

**Figure 8:**
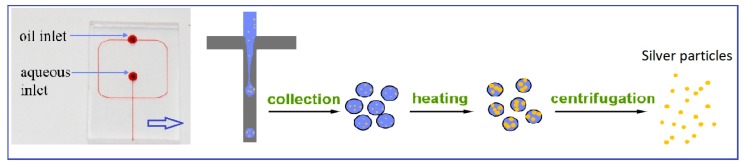
Schematic of the whole synthesis process of silver nanoparticles.

A method to synthesize silver nanoparticles with controlled size and spectral tune ability was developed (Figure 8). In this study, nanoparticles were prepared via a simple seed-mediated growth pathway in microdroplets reactors. The size of silver nanoparticles can be controlled by varying the reaction time and temperature, and the initial concentration of silver nitrate and seeds. The results indicated that the average size of silver nanoparticles increased with increasing reaction time, temperature, and concentration of silver cations, and decreased with increasing seed concentration. Moreover, the effect of different preparation conditions on LSPR wavelength was also investigated. We believe that the method possesses a powerful potential to synthesize other size- and morphology-controlled noble metal in continuous flow microdroplet reactors. These obtained nanoparticles with optimum properties are promising substrates to improve SERS detection efficiency for quantitative analysis.
